# Dilemma of Dilemmas: How Collective and Individual Perspectives Can Clarify the Size Dilemma in Voluntary Linear Public Goods Dilemmas

**DOI:** 10.1371/journal.pone.0120379

**Published:** 2015-03-23

**Authors:** Daniel B. Shank, Yoshihisa Kashima, Saam Saber, Thomas Gale, Michael Kirley

**Affiliations:** 1 Melbourne School of Psychological Sciences, The University of Melbourne, Victoria, Australia; 2 Department of Computing and Information Systems, The University of Melbourne, Victoria, Australia; Lanzhou University, CHINA

## Abstract

Empirical findings on public goods dilemmas indicate an unresolved dilemma: that increasing size—the number of people in the dilemma—sometimes increases, decreases, or does not influence cooperation. We clarify this dilemma by first classifying public goods dilemma properties that specify individual outcomes as individual properties (e.g., Marginal Per Capita Return) and group outcomes as group properties (e.g., public good multiplier), mathematically showing how only one set of properties can remain constant as the dilemma size increases. Underpinning decision-making regarding individual and group properties, we propose that individuals are motivated by both individual and group preferences based on a theory of collective rationality. We use Van Lange's integrated model of social value orientations to operationalize these preferences as an amalgamation of outcomes for self, outcomes for others, and equality of outcomes. Based on this model, we then predict how the public good's benefit and size, combined with controlling individual versus group properties, produce different levels of cooperation in public goods dilemmas. A two (low vs. high benefit) by three (2-person baseline vs. 5-person holding constant individual properties vs. 5-person holding constant group properties) factorial experiment (group n = 99; participant n = 390) confirms our hypotheses. The results indicate that when holding constant group properties, size decreases cooperation. Yet when holding constant individual properties, size increases cooperation when benefit is low and does not affect cooperation when benefit is high. Using agent-based simulations of individual and group preferences vis-à-vis the integrative model, we fit a weighted simulation model to the empirical data. This fitted model is sufficient to reproduce the empirical results, but only when both individual (self-interest) and group (other-interest and equality) preference are included. Our research contributes to understanding how people's motivations and behaviors within public goods dilemmas interact with the properties of the dilemma to lead to collective outcomes.

## Introduction

Many human endeavors are neither accomplished nor enjoyed individually, but often people come together and voluntarily cooperate to produce a public good that benefits more than the individual. These include a range of large- and small-scale projects such as protecting the environment, electing officials, building libraries, parks, and infrastructure, maintaining charitable organizations, producing collaborative research, keeping a clean house, and competing in a multiplayer game or sport. Individuals often find themselves in a social dilemma with regard to public goods in that they would like to enjoy the benefits of the public good (e.g., a clean environment or a clean house), but contributing to it costs them resources such as time, money, and effort. Social dilemma situations form when individuals receive better outcomes for not contributing compared to contributing regardless of what others do, yet all individuals receive worse outcomes if no one contributes compared to everyone contributing [1]. Public goods dilemmas are a type of social dilemma where all can benefit from a collective resource, a public good, regardless of who contributed to it [2]. Therefore, contributing to the public good (cooperating) is never as personally beneficial to one’s own outcomes; however, not contributing (defecting), while personally yielding more *individual benefit*, leads to less *collective benefit* [2, 3].

A fundamental feature of public goods dilemmas is the number of people involved, which can range from two (e.g., household) to billions (e.g., environment). This difference in size can alter the level of cooperation; however how it is altered is an unresolved dilemma as different empirical studies indicate either positive, negative, or no effects, as we review later. Explanations of size effects in public goods games generally fall into two categories. One category draws on the incentive or payoff structure, that is characteristics of the dilemma [4–6], whereas the other category draws on other social psychological factors such as self-efficacy [7], norm-enforcement [8], monitoring [9], punishment [10] and framing [11]. We explore the both types of explanations for the effect of size, proposing that, psychologically, people may reason from both an individual and group perspective and that this reasoning clarifies why different payoff structures have produced the size dilemma.

Because individual and group actions are interrelated in public goods dilemmas, a person can conceptualize decision-making, behavior, and outcomes from an individual or group perspective, i.e., in terms of the dilemma’s properties applied to the individual or group. Whereas the individual actor’s perspective is a traditional foundation for theorizing [12, 13], recent work suggests that people also reason at a group or team level, often referred to as collective rationality [14–17]. Herein, we use Van Lange’s integrative model of social value orientations [18] which accounts for individual and group perspectives [14] by integrating preferences for self, others, and equality into one weighted model of decision-making.

A number of researchers have investigated group size in public goods dilemmas [4, 5, 19, 20], but, we argue, that an implicit assumption about an individual or group perspective was embedded in their implementation of the public goods dilemma leading to the range of empirical results. Therefore, we extend Van Lange’s integrative model to public goods dilemmas, demonstrating how the simultaneous consideration of individual and group perspectives can clarify this size dilemma. We show that the divergent specifications used within other studies can be simplified to varying differences in overall benefit—that is the value of cooperating—and that this benefit changes with size based on properties affiliated with either an individual or group perspective. We conduct an experiment which crosses low or high benefit with the type of property held constant as size increases, confirming the different relationships between size and cooperation found in previous research. Then, to show that the integrative model can reproduce the empirical results across the different property specifications, we use an agent-based simulation with agents that combined individual and group preferences to determine their behavior. Whereas matching the empirical results shows that the integrative model is sufficient to account for the different specifications, the parameter estimation also is suggestive of how individuals might integrate individual and group perspectives.

## The Multiplayer Public Goods Dilemma

A voluntary linear multiple-player public goods dilemma differs from common resource dilemmas and step-function public goods dilemmas in several important ways. Public goods dilemmas focus on people pooling resources or effort to provide a good for all, whereas common resource dilemmas relate to taking resources from a common pool. For linear public goods dilemmas every contribution creates additional benefit, whereas step-function public goods dilemmas the public good is provided only if a particular step threshold is obtained. Example of step-function dilemmas include fund-raising to build a new library or a certain number of people needed to serve on a committee [21], whereas examples of linear public goods include the environment, a clean house, a potluck dinner, or reviews on a website such that additional contributions continue to increase the benefit of that good for everyone.

We defined a linear public goods dilemma such that the dilemma’s participants have a binary choice to contribute (cooperate) or not contribute (defect) each round over multiple rounds of interaction. For each round, an individual’s outcome is defined by the following:

$$\mathrm{outcome}=\{\begin{array}{ll} \frac{cz\alpha }{n} & \mathrm{if}\;c_{s}=1\left(contributes\right)\\ \frac{cz\alpha }{n}+z & \mathrm{if}\;c_{s}=0\left(\mathrm{does not contribute}\right) \end{array}$$ (1)

where *n* is the number of total participants (size), *c* is the total number of contributors (*c*_*s*_ is 1 if self contributes; *c*_*O*_ is number of others who contribute, such that *c* = *c*_*s*_ +*c*_*o*_), *z* is each participant’s endowment, and *α* is the dilemma multiplier. Explained discursively, each renewed endowment of *z* that is contributed to the group is multiplied by α, then divided among the group members *n* regardless of who contributed, whereas any endowment *z* not contributed is kept towards one’s own outcome. Breaking down the effects based on what a participant can control, on any given round the portion that participants receive from their own decision is $\frac{z\alpha }{n}$ for contributing (if *c*_*S*_ = *1* then *c* is 1 greater) or *z* for not contributing (*c*_*S*_ = *0*). Therefore, a participant’s temptation to defect [5], the profit one will earn by not contributing, is $z-\frac{z\alpha }{n}$. Furthermore, participants earn $\frac{z\alpha }{n}\;$ per additional group member, *c*_*O*_, that contributes, meaning a potential earnings for full cooperation is *zα* when *c = n*. The value $\frac{z\alpha }{n}$ must always remain less than *z* to be classified as a public goods dilemma; otherwise one’s payout would be largest by always contributing to the public good. Consequently, the multiplier is constrained to *1 ≤ α < n*. For an illustration, suppose *z = 100*, *α = 1*.*6*, and *n = 2*. Then the outcome a participant will earn each round would be 160 if both contribute (*c*_*S*_ = *1; c*_*O*_ = *1*), 100 if neither contribute (*c*_*S*_ = *0; c*_*O*_ = *0*), 180 if only the other player contributes (*c*_*S*_ = *0; c*_*O*_ = *1*), or 80 if only the participant contributes (*c*_*S*_ = *1; c*_*O*_ = *0*).

### Properties that Vary with Group Size

A central issue for investigating group size in voluntary public goods dilemmas involves two types of properties from the dilemma that vary differently with the group size. The first type relates to the entire group’s calculus or outcomes for the social dilemma, such as the dilemma’s multiplier or the earning potential if all participants cooperate. These group properties represent the group-focused aspects of the dilemma, addressing the question: *what is most beneficial for the entire group*? Therefore an actor with only a group perspective would interpret the dilemma in terms of these group properties. The second type of property relates to the individual participants’ reward calculus and outcomes, such as their temptation to defect or marginal per capita return (MPCR). The MPCR, $\frac{\alpha }{n}$, is a ratio of the individuals’ marginal benefit, $\frac{z\alpha }{n}$, to the individuals’ marginal cost, *z*, for contributing. These individual properties remain structured by the overall specification of the public goods dilemma, but are psychologically understood from the vantage of one individual asking the question: *what is most beneficial for me*? Therefore, an actor with only an individual perspective would interpret the dilemma using these individual properties.

**Fig 1:**
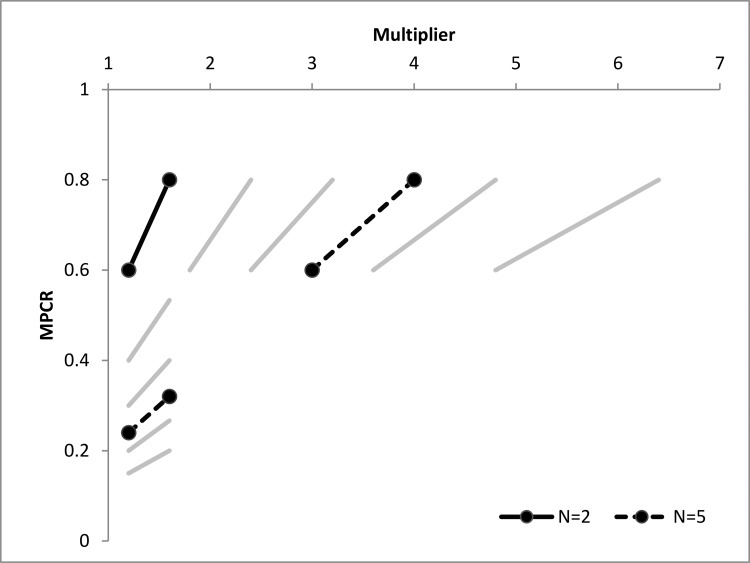
The relationship of the multiplier and MPCR for different sized groups, holding constant the multiplier at 1.2 and 1.6 or holding constant the MPCR at 0.6 and 0.8. The dark lines are for N = 2 and N = 5, and lighter lines are for N = 3, N = 4, N = 6, and N = 8. Circle endpoints indicate the six conditions parameters for this study.

It is critical to note that either the group or individual properties can be held constant as the dilemma size increases, but not both. The multiplier, *α*, and earning potential for full cooperation, *zα*, do not contain the group size variable *n*. In contrast, the marginal per capita return, $\frac{\alpha }{n}$, and the temptation to defect, $z-\frac{z\alpha }{n}$, both vary based on the group size. Examining the formulas for these different properties, we see the simplest group and individual properties are the multiplier and the *MPCR* because they exclude the endowment. Whereas the endowment is important for baseline levels of cooperation behavior, if it is held constant it is not essential for a comparison of how group and individual properties differ by dilemma size (see Table 1). Note that $MPCR\;=\frac{\alpha }{n}$ and therefore the individual property MPCR decreases with size if the multiplier, a group property, is held constant. However, the same equation rewritten as α = MPCR * n shows that group properties such as the multiplier α increase with size when the MPCR, an individual property, is held constant (see Fig. 1 for their relationship and S1 Fig. and S2 Fig. for a comparison of the multiplier and MPCR).

**Table 1:** Four properties of voluntary public goods dilemmas.

	Properties without Endowment		Properties with Endowment	
Group Properties	Dilemma Multiplier	*α*	Earnings for Full Cooperation	*zα*
Individual Properties	Marginal Per Capita Return (MPCR)	$\frac{\alpha \;}{n}$	Temptation to Defect	*z—$\frac{z\alpha }{n}$*

### Individual and Group Perspectives and Properties

Whereas it is often assumed that people reason from an individual perspective to increase their individual gain or decrease their individual loss, contemporary theorizing suggests that people also reason from a team or collective viewpoint, namely, act to increase the collective gain, to decrease the collective loss, or to increase equality [14, 15, 17]. Colman *et al*. [15] suggests that *collective rationality*, that is a rationality that occurs individually but includes reasoning about a team or group, is fundamental in many decision making contexts. Applying it to game theory and vignettes, they find that collective rationality predicts a large percentage of individual’s decisions, in some cases better than an individual rationality, or self-interest.

In a similar vein, Van Lange [22] has proposed an integrative model of social value orientations whereby prosocially oriented individuals attempt to not only jointly maximize their own and other’s outcomes, but also the equality of outcomes as shown in Equation 2.

$$\mathrm{Utility}=W_{1}(\mathrm{Outcomes for Self})+W_{2}(\mathrm{Outcomes for Other})+W_{3}(\mathrm{Equality in outcomes})$$ (2)

This model was supported empirically in prosocials’ decision making [22]. Because Coleman *et al*.’s standard for collective rationality was maximizing joint benefit [16], Van Lange [14] showed that this integrative model can account for the findings of Colman *et al*.’s collective rationality. Joint benefit is simply an equal focus on the outcomes to self and others, or applying *W*_*1*_ = *W*_*2*_ = .*5* and *W*_*3*_ = *0* to the integrative model equation. Therefore, we note that the integrative model represents collective rationality in terms of maximum joint benefit and extends it to include other group-based preferences, namely equality. Equality is an important factor for considering collective rationality because of its presence in social value orientations [22] and as an explanation for group behavior [23].

Using the integrative model to capture both individual and group preferences, we reason about how its different components could alter behavior in public goods dilemmas that vary on size and individual or group properties. In public goods dilemmas the best *Outcomes for Self* is always produced by defecting, however the strength of this varies based on the dilemma’s individual properties. A higher Marginal Per Capita Return, for example, means that cooperative behavior returns a larger proportion of the contribution, thereby mitigating the appeal of defection. Conversely, the *Outcomes for Others* (which we pluralize to account for multiple other participants) is always maximized by contributing in a public goods dilemma, yet the strength of this also varies based on individual properties of the dilemma. The greater the MPCR, the more others benefit from a participant’s cooperation. Whereas maximizing outcomes for oneself versus others evoke opposite strategies (e.g., cooperation or defection), a higher MPCR enhances the utility of cooperation in both cases. Therefore, because MPCR decreases with size when group properties are held constant, we expect (H1) cooperation should decrease with group size if the group properties are held constant, yet (H2) cooperation should not change with group size if the individual properties are held constant.

Maximizing equality in outcomes suggests a preference for full cooperation or full defection as these produce the same—that is equal—outcomes for everyone. For a 2-person group, an individual’s decision always alters the *Equality of Outcomes* because matching the other participant’s choice with cooperation or defection produces full equality and opposing it produces maximum inequality (i.e., the outcomes of one defector and one cooperator). For a larger group, say five people, a participant’s decision to cooperate or defect has different effects on equality outcomes depending on the decisions of others, as we later mathematically demonstrate. If two players cooperate and the other two defect, the inequality of the outcomes cannot be reduced by either cooperation or defection (i.e., either there will be two cooperators and three defectors, or vice versa). However, if four players cooperate, then cooperation produces fully equal outcomes whereas defection produces less equal outcomes. This line of reasoning leads to the counterintuitive conclusion that pursuing equality among outcomes can lead to polarized collective decisions. It also indicates that that equality will have a stronger polarization effect for smaller groups and weaker polarization effect for larger groups.

Whether equality concerns will push a group towards full defection or full cooperation depend on the base levels of cooperation. In groups where there is a higher benefit from the public good, the equality of outcomes will be more likely to promote cooperation, whereas in groups with a lower benefit from the public good the equality of outcomes will be more likely to promote defection. Therefore, we expect (H3) cooperation levels should differ between levels of benefit and be most pronounced in the 2-person groups. As we will see next, the empirical findings appear to be consistent with these theoretical hypotheses.

### Previous Research on Properties and Group Size

The literature on linear public goods dilemmas has found that size can increase [4, 6] or decrease [20, 24, 25] cooperation, sometimes depending on the condition [5, 26]. Field study data has also shown under different conditions group size increase [27, 28] or does not influence [29] contributions and cooperation. Because of the variation in results, a meta-analysis of linear public goods studies found that size did not have a statistically significant impact on contributions [30].

Nonetheless, when group properties are held constant, cooperation decreases with group size. For example, Yamagishi [20] compared group sizes ranging from 2 to 501 while holding the multiplier constant in three experiments. He found that as group size increased, cooperation levels decreased, however this pattern was primarily for groups of smaller sizes below 10. This supports our first hypothesis that cooperation should decrease with group size when group properties are held constant, as well as our third hypothesis that cooperation levels should be most pronounced in smaller size groups.

There is also evidence that when the individual properties are held constant, cooperation increases or does not change with group size. For example, Isaac and Walker [4] compared group sizes of 4 and 10 and held constant the MPCR at .30 or .75. They altered initial endowments by condition so that the complete cooperation earning potential remained constant. They found that when the MPCR was high, size had little effect on cooperation; however cooperation increased with group size when the MPCR was lower. Their conclusion was that at high MPCR, group size no longer matters as it does at low levels. A later study [31] compared groups of size 4 to 100 making a similar conclusion, that at high levels of MPCR, size did not alter cooperation, whereas it did at lower levels of MPCR. Other research [6] supports this position by finding that even differences in extremely low levels of MPCR can alter cooperation in large groups (60 and 100).

Isaac and colleagues interpret their results as cooperation increasing with size at low benefit, but they can be reasonably reinterpreted as cooperation increasing with benefit at small sizes. Similarly, they conclude that under conditions of greater benefit, size does not alter cooperation, which can be reasonably reinterpreted as under conditions of greater size, benefit does not alter cooperation. Therefore, the pattern of findings of this literature dovetails with both our second and third hypothesis. The compatibility of this previous research with our predictions gives support to our argument about connecting individual and group preference with individual and group properties through the integrative model; however none of these experiments examined group size while holding constant each type of property.

## Experiment

We conduct an experiment according to our earlier dilemma specifications crossing group size while either holding individual or group properties constant with high or low public goods benefit. We hold the endowment *z* constant at 100 points for all conditions. We consider the difference between group and individual properties by keeping either the multiplier or MPCR constant as the group size increases (Fig. 1). We create two baseline experimental conditions where the group size is 2. Based on previous literature, one has lower public goods benefit with a multiplier of 1.2 and a MPCR of .60 and the second has a higher benefit with a multiplier of 1.6 and a MPCR of .80. From each of these baseline conditions we create comparison conditions by increasing the group size to 5 either holding the multiplier or the MPCR constant (see Table 2). This research was approved by the Department Human Ethics Advisory Group, Melbourne School of Psychological Sciences, University of Melbourne (#1340741). All participants read a plain language statement detailing the research and checked a consent box as written consent before being allowed to participate.

**Table 2:** Experimental Conditions.

	2-Person	5-Person	
		Multiplier Constant	MPCR Constant
Lower Benefit	Multiplier = 1.2	Multiplier = 1.2	Multiplier = 3.0
	MPCR = .60	MPCR = .24	MPCR = .60
Higher Benefit	Multiplier = 1.6	Multiplier = 1.6	Multiplier = 4.0
	MPCR = .80	MPCR = .32	MPCR = .80

### Participants

US participants on Amazon Mechanical Turk (mturk.com) chose to participate in our study for financial compensation (2.50 USD plus a bonus of approximately 1.00 to 4.00 USD based on points earned). They were then directed to a web-based program especially designed for this research. The instructions explained to participants their two choices of “keeping” (defecting) or “contributing” (cooperating) their endowment of 100 points each round. The program presented a table with the number of points they would earn based on their own contribution choice and others’ contribution choices. This table of point outcomes differed based on the participants’ condition, but was displayed each time the participants selected a choice. Additionally, details about how the table was derived from the public goods dilemma multiplier and the multiplier itself were also presented. To ensure adequate understanding, participants had to correctly answer quiz questions about a table of point outcomes before playing the game.

Participants were assigned to a dilemma group and completed 31 rounds with that group. After all group members made a selection each round, a feedback screen would indicate to each participant their choice, their groups’ total choices (e.g., number who contributed and number who kept their endowment), and the points they earned. No information was presented from previous rounds except the participant’s personal cumulative point total. Participants had no information about the others they were interacting with except the total number who contributed or kept the endowment each round. Additional measurements were collected before and after the dilemma.

To ensure that participants could not be held up indefinitely by others in their group, there was a time limit for each round. If a selection was not submitted within that time the program selected *contribute* for that participant. Participants who failed to select a choice on any three rounds were timed out of the program and we excluded that entire group from our dataset. We continued collecting data until we had at least 15 groups per condition where no participants timed out of the program (99 groups with 390 participants).

## Results

### Excluded Data

Programmed responses accounted for 21 (0.2%) of the responses, but no group had more than the individual’s maximum of two programmed responses in total. Even though programmed responses were always cooperative, there is no indication they had a substantial or systematic impact on our results (see S1 File for further explanation and analysis). Programmed responses are excluded for all analyses. Because the data can be analyzed at multiple levels, we first examine data with groups as the unit of analysis to present an overall picture and simple statistics. Then we examine data with the individual as the unit of analysis using more complex statistics to control for the group-level interdependence. The group-level analyses based on 99 groups do not have the statistical power of the individual analyses based on 390 participants and therefore the group-level analyses are overly conservative.

### Group-Level Analyses

For group-level analyses, cooperation for each group was calculated as the average cooperative decisions across all rounds for all individuals in the group. The two-person public goods groups produced different levels of cooperation based on the benefit level. The lower benefit condition (α = *1*.*2; MPCR* = .*60*) led to less cooperation (.354), whereas the higher benefit conditions (*α* = 1.6; *MPCR* = .80) led to greater cooperation (.629; Means in Table 3), *t*(33) = -2.04, *p* = .050. Additionally, neither condition produced near complete cooperation or defection, thereby allowing an increase in size to either increase or decrease cooperation.

**Table 3:** Counts and group cooperation means by condition.

		2-Person	5-Person	
			Multiplier Constant	MPCR Constant
Lower Benefit	Counts	n_g_ = 18; n_i_ = 36	n_g_ = 17; n_i_ = 85	n_g_ = 16; n_i_ = 80
	Group Cooperation	.354 (.402)	.162 (.119)	.602 (.251)
Higher Benefit	Counts	n_g_ = 17; n_i_ = 34	n_g_ = 16; n_i_ = 80	n_g_ = 15; n_i_ = 75
	Group Cooperation	.629 (.397)	.237 (.161)	.611 (.206)

Note: *n*_*g*_ is the number of groups; *n*_*i*_ is the number of individuals. Standard deviation in parentheses.

**Fig 2:**
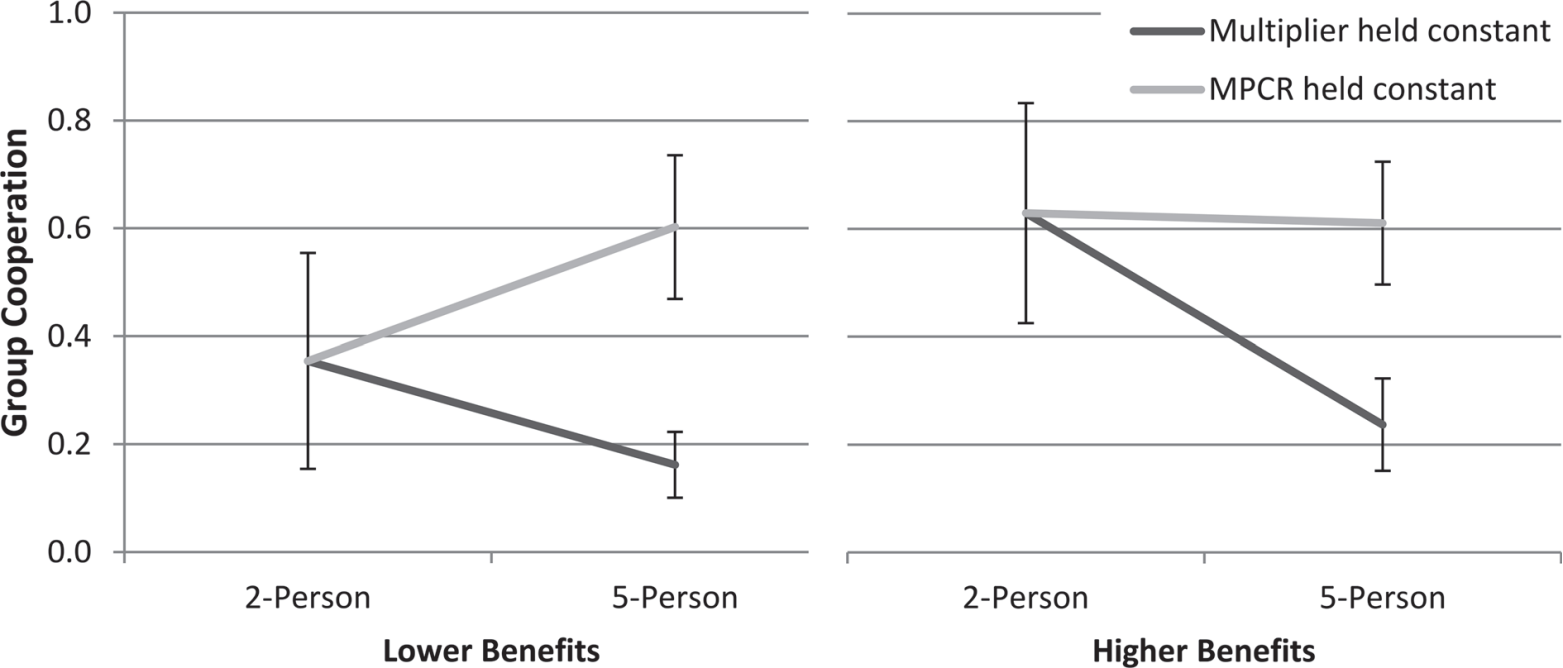
Group cooperation by conditions for 2-Person groups and 5-Person groups where either the Multiplier or MPCR was held constant (with 95% confidence intervals).

Both five-person lower benefit conditions matched the two-person by either holding constant the multiplier or the MPCR (Fig. 2). When the multiplier was held constant the mean cooperation decreased from .354 to .162, which was marginally significant, *t*(20.11) = −1.94, *p* = .066 (equal variances not assumed), whereas when the MPCR was held constant the mean cooperation significantly increased from .354 to .602, *t*(28.83) = 2.19, *p* = .037, equal variances not assumed. Furthermore, the multiplier-constant condition produced more cooperation than the MPCR-constant condition, *t*(21.16) = -6.40, *p* ≤ .001, equal variances not assumed.

The same comparisons were made for the higher benefit conditions. Holding the multiplier constant when increasing the size from two to five people led to cooperation decreasing from .629 to .237, *t*(21.36) = −3.76, *p* ≤ .001 (equal variances not assumed), whereas holding the MPCR constant led to no significant change in cooperation levels from .629 to .611, *t*(24.63) = −.17, *p* = .866, equal variances not assumed. The multiplier-constant condition also had greater cooperation than the MPCR-constant condition, *t*(29) = −5.65, *p* ≤ .001.

To examine the overall effects of condition, we created dummy variables for the benefit level (low or high), and the multiplier-constant conditions and the MPCR-constant conditions (with 2-person groups as the reference). When conducting an ANOVA with the dummy variables and an intercept, all three dummy variables were significant (Table 4: Model 1; all *p*’s ≤ .05) such that high benefit increased cooperation, holding the multiplier constant size negatively affects cooperation, and holding the MPCR constant size positively affects cooperation. When including the interaction effects in the model (Model 2), the multiplier-constant dummy still decreases cooperation (H1), but the other two dummy variables no longer influence cooperation (H2). There is a marginally significant effect, *F*(1,93) = 3.67, *p* = .059, for Benefit X MPCR-constant interaction, suggesting that at low-benefit constant MPCR, size positively affects cooperation; however at higher levels of benefit this increase is no longer present (H3). To determine if these tendencies hold for a more rigorous higher power analysis, we now turn to analyzing the data by individual.

**Table 4:** ANOVA predicting group cooperation by benefit level and size\type.

	Model 1		Model 2	
	F(1, 95)	p	F(1, 93)	p
High Benefit Level	4.744	.032	.350	.555
Multiplier Constant (5-p)^a^	17.448	.000	18.167	.000
MPCR Constant (5-p)^a^	2.845	.032	2.710	.103
High Benefit X Multiplier Constant			2.125	.148
High Benefit X MPCR Constant			3.668	.059

^a^ 2-Person groups are reference

### Individual-Level Analyses

Individual cooperation was calculated as each participant’s average cooperation over all rounds. When analyzing data at the individual level it is important to control for the interdependence between individuals as individuals in the same group can affect each other’s cooperation rates over time. Specifically, the interclass correlation rate is .6336, suggesting that over half of the individual variance in cooperation is explained by group-level parameters (which in this case includes the conditions as groups always functioned within a set of condition parameters).

We conducted mixed models analyses that included group intercepts as a random effect covariant parameter with the design factors as fixed effects. The null model (Table 5: Model 1) indicates the statistical significance of the groups’ intercepts (.091, *Std Error* = .0156), *Wald Z* = 5.819, *p* ≤ .001, and baseline fit statistics. Adding in the condition dummy variables (Model 2), we find similar patterns to our group-level analysis (H1) except that the MPCR-constant dummy variable is only marginally significant (.119) *F* = 3.170, *p* = .078, and remains so in Model 3 (H2). Adding the interaction effects in Model 3, this analysis show the interaction of High Benefit X MPCR-Constant conditions has a negative and significant effect on cooperation (-2.67) *F* = 4.099, *p* = .046. We interpret this to mean that when MPCR is held constant, benefit has a stronger influence on cooperation between 2-person dilemmas than between 5-person dilemmas (H3). Thus, the interaction effect of our third hypothesis qualifies the simple effect of MPCR-constant size changes on cooperation, our second hypothesis.

**Table 5:** Mixed models predicting individual cooperation by group membership, benefit level, and size\type.

	Model 1			Model 2			Model 3		
	Estimate	F/Ward Z^b^	p	Estimate	F/Ward Z^b^	p	Estimate	F/Ward Z^b^	p
**Fixed Effects**									
Intercept	.425	168.516	.000	.376	159.085	.000	.354	162.445	.000
High Benefit Level				.114	4.546	.036	.275	.473	.511
Multiplier Constant (5p)^a^				−.289	19.378	.000	−.192	20.229	.000
MPCR Constant (5p) ^a^				.119	3.170	.078	.248	3.028	.085
High Benefit X Multiplier Constant							−.200	2.366	.128
High Benefit X MPCR Constant							−.267	4.099	.046
**Random Effects**									
Groups’ Intercepts (Covariance)	.0908	5.819	.000	.0549	4.945	.000	.0532	4.853	.000
Residual	.0525	11.976	.000	.0533	11.792	.000	.0533	11.794	.000
**Information Criteria**									
−2 Restricted Log Likelihood	159.051			131.927			132.352		
Schwarz’s Bayesian Criteria (BIC)	170.978			143.839			144.254		

^a^ 2-Person groups are reference

^b^ F statistics are used for fixed effects and Ward Z statistics are used for random effects

### Discussion

Overall, we found some support for all three hypotheses, with the marginal support of the second hypothesis in the individual analyses being due primarily to its qualification by the third hypothesis. Most notably our results were in line with previous studies. First, for the 2 person public goods dilemmas the benefit level was the major predictor of cooperation levels. Consistent with prior work, increased size either promoted more, less, or the same amount of cooperation depending on whether group or individual properties were held constant and benefit level [4, 5, 20]. This interaction between size and benefit dovetails with previous studies that have found at high levels of MPCR group size no longer has discernible influence on cooperation [31]. This was also our surprising and counterintuitive prediction based on reasoning about the dilemma’s properties and the group-preference of equality from the integrated model of social orientations. We now turn to agent-based modeling to further explore whether the group and individual preferences of the integrated model are able to be fit to these empirical results.

## Simulation using a Model of Individual and Group Preferences

We conduct a simulation of agents that uses Van Lange’s [14, 22] integrative model (Equation 2) to show that the pattern of our cooperation results, which accounts for the size dilemma, can be produced using this implementation of individual and group preferences without the need of additional complex assumptions about individual actors. In the integrative model’s weights (*W*) allow it to be reduced to individual preferences such as self-interest (W_1_ = 1, W_2_ = 0, W_3_ = 0), group preferences such as altruism (W_1_ = 0, W_2_ = 1, W_3_ = 0), and combined preferences such as cooperation (W_1_ = .5, W_2_ = .5, W_3_ = 0) or egalitarianism (W_1_ = .5, W_2_ = 0, W_3_ = .5). Any set of estimated weight parameters that are able to reproduce the empirical results will indicate a preference combination sufficient to reproduce our size dilemma conditions.

We created a public goods dilemma simulation in *R* according to the same specifications as our empirical study. For each run of the simulation, agents were selected randomly from our population. Based on the previously reported levels of freeriding behavior in social dilemmas [32], we included free-riders—that is complete defectors—as one-third of the population. The other two-thirds were *mixed motive agents* based on the integrative model. To ensure the simulations were stochastic, every agent on every round had a 10% chance of behaving randomly (50% probability of cooperating).

### Mathematical Specification of the Integrated Model Applied to Linear Public Goods Dilemmas

The mixed motive agents behave randomly on the first round (50% probability of cooperating). Thereafter, they use the utility function to calculate the utility of cooperating or defecting premised on all others making the same decisions as the previous round. Assuming others will make the same choice as before seems to us to be the simplest model of others’ behavior. Based on others’ previous choice, the agent calculates the utility for the current round. If the utility for cooperating exceed the utility for defecting the agent cooperates. *Outcomes for Self* is operationalized as one’s own points earned on one round according to Equation 1. Because all individual outcomes on a given round are either $\frac{cz\alpha }{n}\;$ for cooperation or $\frac{cz\alpha }{n}+z$ for defection, Equation 1 can be rewritten as one equation:

$$Outcomes\;for\;Self\;=\frac{cz\alpha }{n}+(1-\;c_{S})z$$ (3)

The difference in *Outcomes for Self* from switching from cooperation to defection, meaning *c*_*S*_ = 0 and therefore *c* decreases by 1, is always an increase by the Temptation to Defect, $z-\frac{z\alpha }{n}$. *Outcomes for Others* is calculated as the average outcome all other players receive per round:

$$Outcomes\;for\;Others\;=\;\frac{cz\alpha }{n}+\frac{(n-1-c_{O})z}{n-1}$$ (4)

In our empirical study and most public goods research with groups larger than 2, participants are not aware of the contributions or cooperation levels of specific individuals. Instead they can see a total level of cooperation and from this it is easy to calculate the points received by others, as everyone in the dilemma receives either $\frac{cz\alpha }{n}\;$ for cooperation or $\frac{cz\alpha }{n}+z\;$ for defection (Equation 1). While the average points others received from the dilemma can vary greatly, the utility difference for others between a participant’s choice to defect or cooperate is always $\frac{z\alpha }{n}$, which is the *MPCR* x the endowment. Therefore, when switching from cooperation to defection—that is *c* decreases by 1, but *C*_*O*_ does not change—*Outcomes for Others* decreases by $\frac{z\alpha }{n}$ points (Equation 4).

Finally, *Equality in Outcomes* is calculated as the difference between one’s own outcome and the average outcome of all other players (Equations 3 and 4). The negative absolute value of this difference is added to the utility equation, so full equality in outcomes is 0, with negative numbers representing more inequality:

$$Equality\;of\;Outcomes\;=-\left|\left(\frac{cz\alpha }{n}+\left(1-c_{S}\right)z\right)-\left(\frac{cz\alpha }{n}+\frac{\left(n-1-c_{O}\right)z}{n-1}\right)\right|=-\left|\;z\left(1-c_{S}-\;\frac{n-1-c_{O}}{n-1}\right)\right|$$ (5)

When everyone cooperates, c_*S*_ = *1; c*_*O*_ = *n-1*, or defects, c_*S*_ = *0; c*_*O*_ = *0*, *Equality of Outcomes* is 0, but when the individual defects and the all others cooperate (or vice versa), c_*S*_ = *0; c*_*O*_ = *n-1*, *Equality of Outcomes* becomes the negative of the endowment-*z* (see S3 Fig. for a full example).

We want to note some interesting properties about these functions. The utility difference between cooperation and defection for both self and others outcomes does not change dynamically within the dilemma. The temptation to defect and the MPCR X the endowment will be the same throughout a particular dilemma regardless of the decisions of any player in it. That means that individuals relying only on these strategies should not deviate in their decisions throughout the session. While this may trivialize simulation results based on only these two functions, the model itself still has profound implications for the overall levels of cooperation. For example, if outcomes to self and others are equally weighted, then the agents in the multiplier-constant five person conditions (those with MPCRs < .5) will choose full defection, whereas the agents in the other four conditions will choose full cooperation. The response to the parameters of the dilemma (Table 6) can be altered with a different integrative model weighting scheme, but will not be affected by players’ round-to-round decisions.

**Table 6:** Experimental conditions with values for outcome to self and others.

	2-Person	5-Person	
		Multiplier Constant	MPCR Constant
Lower Benefit	Multiplier = 1.2	Multiplier = 1.2	Multiplier = 3.0
	MPCR = .60	MPCR = .24	MPCR = .60
	Self (Temptation to Defect): 40	Self (Temptation to Defect): 76	Self (Temptation to Defect): 40
	Others: 60	Others: 24	Others: 60
Higher Benefit	Multiplier = 1.6	Multiplier = 1.6	Multiplier = 4.0
	MPCR = .80	MPCR = .32	MPCR = .80
	Self (Temptation to Defect): 20	Self (Temptation to Defect): 68	Self (Temptation to Defect): 20
	Others: 80	Others: 32	Others: 80

*Equality of Outcomes*, however, can dynamically change over rounds within the dilemma and can additionally create threshold points based on others behavior. As more players cooperate in a dilemma, the utility for the *Equality of Outcomes* is improved by additional cooperation and as more players defect, the utility of *Equality of Outcomes* is improved by additional defection. In this way, since one cannot alter the outcome of specific others, this equality function simply suggests going along with the crowd in either defecting or cooperating. In five-person dilemmas this creates a threshold when two others cooperate and two others defect (S3 Fig.) in which there is no equality advantage for selecting one option over the other. Whereas the threshold points are stable within a dilemma’s specific parameters, combining it with the self and other interest in the weighted utility function can create alternate threshold points. For this we conduct simulations.

### Simulation Results

We created 67 conditions by considering all weight combinations of *W*_*1*_ *+ W*_*2*_ *+ W*_*3*_ = *1* where all *W’s* ∈ {0,.1,.2,.3,.4,.5,. 6,.7,.8,.9,.1} plus a balanced weighting condition (all $W’s=.3\overset{-}{3}$) allowing for us to examine a range of utility weight combinations. We conducted 500 public good dilemma simulation trials for each of 402 conditions: 67 weight conditions * 6 empirical experimental conditions (Table 2). The results indicate that only one weight condition, *W*_*1*_ = .*2*, *W*_*2*_ = .*5*, *W*_*3*_ = .*3*, reproduced the cooperation level pattern seen in the empirical results (Table 7). The other weight conditions, including the more pure models (e.g., purely self-interested) do not reproduce the empirical pattern (Table 7).

**Table 7:** Simulation results compared with empirical results.

		2-Person		5-Person				Weights		
				Multiplier Constant		MPCR Constant				
		Low Benefit	High Benefit	Low Benefit	High Benefit	Low Benefit	High Benefit	W_1_	W_2_	W_3_
Empirical Results		.354	.629	.162	.237	.602	.611			
Simulated Results	Purely Self-Interested	.060	.059	.061	.060	.059	.061	1	0	0
	Purely Other-Interested	.640	.640	.631	.642	.639	.630	0	1	0
	Purely Equality-Interested	.258	.259	.071	.078	.079	.090	0	0	1
	Others & Equality-Interested	.247	.261	.335	.304	.616	.597	0	.5	.5
	Cooperative	.623	.633	.061	.060	.638	.640	.5	.5	0
	Egalitarian	.259	.240	.059	.058	.083	.084	.5	0	.5
	Balanced Weighting	.271	.268	.061	.085	.304	.601	.33	.33	.33
	Best Fit Model	.248	.616	.089	.308	.618	.644	.2	.5	.3

We conducted several variations of the simulations to ascertain the robustness of the model. First, we simulated all conditions with a population of only mixed motive agents, that is we excluded the 1/3 defectors from the population. Second, we repeated the simulation of all conditions with a random normal distribution term (M = 0; SD = 50) added to the integrated utility function allowing the utility of cooperation versus defection to be selected probabilistically. Third, we simulated all conditions with a random normal distribution term (M = 0; SD = .1) added to all three weights of each individual agent. In all three variations the *W*_*1*_ = .*2*, *W*_*2*_ = .*5*, *W*_*3*_ = .*3* weight combination continued to produce the closest pattern to the empirical results, albeit with much higher cooperation levels in the no-defector simulation (S1 Table). Fourth, we conducted simulations using the parameters from the best fit model (*W*_*1*_ = .*2*, *W*_*2*_ = .*5*, *W*_*3*_ = .*3)* with agent memory adapted from Kashima, Woolcock, and Kashima [33]. The temporal utility function, U_M_, at time t is *U*_*M*,*t*_ = *(W*_*M*_)(*U*_*t*_) + (*1-W*_*M*_)(*U*_*M*,*t-1*_) where *W*_*M*_ is the weight of utility for the most recent outcome of the game. We varied *W*_*M*_ from .1 to .9 in steps of .1, and found that these varied only slightly and all replicated the pattern in the empirical data with *W*_*M*_ = .*8* producing the closest match (S1 Table). Finally, we conducted 10 trials of a genetic algorithm to optimize these different robustness parameters (S2 File), and found that the two best solutions include (*W*_*1*_ *≈* .*2*, *W*_*2*_ *≈* .*5*, *W*_*3*_ *≈* .*3)* with low memory (*W*_*M*_) and ∼10 percent random behavior and (*W*_*1*_ *≈* .*35*, *W*_*2*_ *≈* .*45*, *W*_*3*_ *≈* .*2)* with no memory and ∼36 percent random behavior (Table B in S2 File). As the first of these is nearly identical to our previous best solution, this gives us further confidence in this parameter selection.

The initial simulations and the subsequent tests of robustness indicate to us the integrative model without additional assumptions is sufficient to produce the results we observe empirically. Furthermore, the weighting scheme that produced the closest match to empirical results suggests decision-making that accounts for both individual and group preferences, including outcomes to self and others as well as equality of outcomes. *Outcomes for Self* received the smallest weight of all three parameters; however, these weights may be only appropriate to with regard to the parameters selected for these experimental conditions.

### Further Explanation: Collective Response to Low Levels of Cooperation

One phenomenon present in the empirical data is a difference in how groups collectively respond to low levels of cooperation. Because cooperation is relatively more favorable for both *Outcomes to Self* and *Outcomes to Others* as MPCR increases, groups with higher MPCR should return to higher levels of cooperation after dropping to a lower level. Comparing all four 5-person group conditions, we observe patterns in which group with higher MPCRs reach lower levels of cooperation less often and when they do immediately return to higher levels of cooperation, whereas groups with lower MPCRs always reach with low levels of cooperation and rarely increase it afterwards. To examine this response pattern we counted all 5-person groups which had at least one round where the number of contributors was 0 or 1. The first of these rounds that occur we refer to as the drop point round, as it must either be a drop from a higher level of contribution unless it is the first round of interaction. For cases that have the drop point round, the next round is a chance to rebound. On this round we find that in the groups with MPCRs of .24 and .34 have average contributions by less than one person, .71 and .75 people, respectively, indicating a failure to rebound. In contrast, the groups with MPCRs of .60 and .80 obtain an average of 1.67 and 2.33 people contributing, respectively, indicating a rebound from the drop point. We selected the drop point of 0 or only 1 player cooperating because it represents—for at least that round—a clear dominance of self-interest. Yet if multiple people contribute in the following round it indicates both collective rationality—that people are behaving motivated by group preferences—as well as collective action, that people while individually making choices produce a group-level result.

We compare this empirical trend to our simulated data from the best fit model (Table 8). The proportion that contains a drop-point round across all conditions is lower for the simulated data than for the empirical data. Also compared to the empirical data, the simulated data indicated even fewer contributors in a rebound round for the low MPCR conditions (around .25) while a similar number of contributors for the high MPCR conditions. Most importantly, it accurately predicts whether there will be a rebound or not after the drop-points in terms of whether the number of contributors exceeds 1. Whereas this is not proof of a mechanism, it does suggest how a model of individual and group preferences in addition to reproducing levels in overall cooperation levels can also reproduce collective action.

**Table 8:** Drop point and rebound rounds^a^ effects for groups in the empirical and simulated data.

		5-Person			
		Multiplier Constant		MPCR Constant	
		Low Benefit	High Benefit	Low Benefit	High Benefit
Empirical Data	Percentage that reached a drop point round	100 (17/17)	100 (16/16)	56.3 (9/16)	60.0 (9/15)
	Average number of rebound round contributors	.71	.75	1.67	2.33
Simulated Data	Percentage that reached a drop point round	98.2 (491/500)	71.2 (356/500)	49.00 (245/500)	51.80 (259/500)
	Average number of rebound round contributors	.25	.24	1.50	2.77

^a^ A drop point round is the first round with 0 or 1 cooperators; the rebound round is the next one.

## General Discussion

This paper has two major contributions. First, we clarified the size dilemma in public goods research: that the size of the group sometimes increases, sometimes decreases, and sometimes does not affect cooperation levels. We took a three-pronged approach to addressing this issue. (1) We classified the properties used in public goods dilemmas as individual (MPCR, Temptation to Defect) and group (Multiplier, Earnings for full cooperation) properties, showing the relationship of those property types to group size and cooperation levels. (2) We connected those properties to individual and group preferences and ultimately to Van Lange’s integrated model of social value orientations both through hypothesis testing and simulations. (3) We experimentally tested and compared both holding individual and holding group properties constant while size varies. This one experiment with a limited number of conditions and groups resulting in several results close to the traditional significance cut off of .05 should be verified by similar future studies. However, our confidence in the results is bolstered given the group-level analyses, individual-level analyses, and simulations converged in line with the theoretical hypotheses and prior literature.

Consistent with our predictions and the previous literature, holding group properties constant decreased cooperation with size, whereas holding individual properties constant did not affect cooperation for public goods dilemmas with a higher level of benefit. For a lower level of benefit and constant individual properties, size increased cooperation which is in line with our counterintuitive hypothesis about equality concerns leading to polarization in smaller groups. Alternatively, this could be interpreted as a ceiling effect, where lower benefit groups increased cooperation with size when individual properties were held constant, but higher benefit groups did hit a cooperation ceiling, potentially caused by a percentage of defectors in the population [32]. Both explanations are reasonable given this data, but the former is theoretically derived from the integrative model of social orientations.

Our second major contribution was applying an explanation of individual decision making to public goods dilemmas based on current research in collective rationality or team-based reasoning, which is reasoning about group preferences in addition to individual preferences. We implemented this theoretical perspective in terms of Van Lange’s integrative model where individuals attend to and are motivated by a combination of self-interest, interest for others, and equality for all. Van Lange has shown that this model appropriately represents the behaviors and expectations of prosocial individuals [22] and is a sufficient explanation for collective rationality [14]. Applying this model to public goods dilemmas of different sizes, we find that the inclusion of an equality term leads to novel predictions as well as dynamic decision making. Not only are predictions based on this model consistent with our results, but the agent-based simulation further confirms that the outcomes and processes from the model to the data are coherent. Using agent based modeling rooted in a theoretical basis and with an empirical benchmark can enhance our understanding of an issue [34], in this case connecting people’s motivations with their individual and collective action, and interactions, within public goods dilemmas.

In regards to continuing work in the area of public goods dilemmas we hope our parameterization will sensitize other researchers to the interrelation between size, properties, and cooperation levels. Our finding of an interaction effect between the change in size and benefit level indicate to some degree that changing the size of a public goods dilemma alters it in fundamental ways instead of always having a simple orthogonal effect. Furthermore, our study shows how the size-based individual and group properties alter cooperation for the particular parameterization we chose. Future research should consider different formulations of the public goods dilemma, such as step-function, one-shot, partial contribution dilemmas, and groups larger than five. Groups of two and five, while substantively different from each other, are relatively small and therefore the explanations we proposed are limited in that they may not scale to larger groups including the largest groups, such as societies. Research has shown that large groups have a pronounced effect on cooperation, but above a certain point there are few effects of further increases in size on cooperation [4, 5, 20, 26, 31].

Whereas we presented individual’s motivation *vis-à-vis* individual and group preferences as a sufficient explanation, it is neither exclusive nor complete. We hope this research will spawn comparisons of competing and complementary explanations. Does collective rationality better explain differences in social dilemmas, especially enigmas in social dilemmas such as the size problem, than explanations based on affective, learning, efficacy, or normative processes? Collective rationality, focused on motivation for decision making, could also be combined with other explanations that detail the mechanisms within one’s decision-making process. A limitation of this study was that we did not measure individual-level properties such as social value orientations [18, 33] or intentions to cooperate. This data would be fundamental to tease apart the individual decision making process in social dilemma settings, and to explore the presence and ordering of potential structural, cognitive, affective and behavioral mechanisms.

The extant literature indicates several possible mechanisms that could function separately or in tandem with collective rationality. In step-level dilemmas, self-efficacy and collective-efficacy decrease with group size as people feel like their contribution matters less and therefore it justifies their uncooperative actions [7, 35], however it unclear if this would generalize to linear public goods dilemmas [21]. Against the rationality of self-interested behavior, social norms develop in situations of collective action [36] which then can facilitate collective behavior through cohesion or sanctioning [37, 38]. Either self-efficacy or social norms could function as a mechanism by which individual and group preferences are enacted, or serve as alternative explanations to preferences based on social value orientations. For example, do individuals who feel empowered focus more on collective outcomes or do collective preferences lead to behavior which enhances self-efficacy? Or does size trigger a lower level of self-efficacy [7], which then changes individual and collective preferences irrespective of the other dilemma properties? We leave it up to future research to see how these and other internal mechanisms might interplay with individual and collective preferences.

Our simulated results spotlight additional research questions. For instance, how do social value orientations differently operate in situations with multiple others? Is prosociality directed toward multiple others individually, to the group as a whole, or is it conditioned on the separability of other’s contributions? We implemented our agent-based model with all other players as a single collective, thereby not increasing the weight of the prosocial terms for additional players. However, one could imagine a pure altruist being more interested in helping the most people, as opposed to helping one individual most. As size within public goods dilemmas is differently structured according to group and individual parameters, size within collective rationality could be applied with different formulations.

A related limitation of our study involves our smallest group containing two people only, whereby the *collective* outside of oneself is only one other person. To fully understand the implications of collective rationality in multiple actor dilemmas it is necessary to recognize how joint outcomes or equality are interpreted for a “collective” of one other person versus a group collective in which the individual members are less separable [39]. Georg Simmel perhaps was the first to suggest the fundamental difference between a dyad, where *groupness* is fully dependent on each person, and a triad, where the group can exist without any other single individual. Similarly, as others have noted [19], a social dilemma of two people compared to those with more than two people can lead to different outcomes. Both the *Outcome for Others* and the *Equality of Outcomes* depend on others, meaning when the total group is greater than two people, these *others’* properties are combinations of multiple people’s behaviors, not just a single individual’s behavior. Future research should consider this in more detail.

In conclusion, this research highlights important issues of theoretical and practical concern. Theoretically, the application of social value orientations into game theoretic dilemmas is not novel, but integration of collectively oriented social values through team-based reasoning introduces a rationality that is not only interesting, but contributes to extant issues. One such issue is the *dilemma of dilemmas* that is the dilemma of the divergent specifications and findings regarding size and public goods dilemmas. Our specification and findings have illuminated this issue and hopefully will encourage researchers across disciplines to carefully specify public goods dilemmas in light of both individual and group properties. Practical applications from this line of research abound as a fundamental difference between the sizes of many real-world public goods dilemmas. An implication for the largest dilemmas, such as the preservation of the natural environment, includes not only focusing on the personal benefits which are often minimal, but also others’ benefits and the equality of them. An integrated focus on all these concepts may be more effective than focusing on one concept to the neglect of the others.

## Supporting Information

S1 Data Supporting data. (SAV)

S1 Fig Marginal Per Capita Return by different sizes of public goods dilemma and different multipliers (α). Because multipliers are constrained from 1 < α < *n*, we have shown four values to cover the range of α when n = 2.
(PDF)

S2 Fig Multiplier values by different sizes of public goods dilemma and different Marginal Per Capita Return values. MPCR values are always between 0 and 1, but for a 2-person public goods dilemma, the MPCR must remain about .5, so we have shown four values to cover the range of .5 to 1.
(PDF)

S3 Fig
*Equality of Outcomes* equation, $-\left|z\left(1-c_{S}-\frac{n-1-c_{O}}{n-1}\right)\right|$, for *n = 5*, *z = 100*, for varying if the individual cooperates, c_*S*_, and how many other players cooperate, c_*O*_.Note that complete equality is 0.(PDF)

S1 File Missing Responses. (PDF)

S2 File Genetic Algorithm Optimization. (PDF)

S1 Table Robustness checks for the simulated results: the models that match the empirical result pattern. (PDF)

## References

[1] Dawes RM. Social dilemmas. Annual Review of Psychology. 1980;31(1):169–93.

[2] Kollock P. Social dilemmas: The anatomy of cooperation. Annual Review of Sociology. 1998:183–214.

[3] Van Lange PA, Joireman J, Parks CD, Van Dijk E. The psychology of social dilemmas: A review. Organizational Behavior and Human Decision Processes. 2013;120(2):125–41.

[4] Isaac RM, Walker JM. Group size effects in public goods provision: The voluntary contributions mechanism. The Quarterly Journal of Economics. 1988;103(1):179–99.

[5] Bonacich P, Shure GH, Kahan JP, Meeker RJ. Cooperation and group size in the n-person prisoners' dilemma. Journal of Conflict Resolution. 1976;20(4):687–706.

[6] Weimann J, Keser C, Stahr C. Public-good experiments with large groups2012.

[7] Kerr NL. Illusions of efficacy: The effects of group size on perceived efficacy in social dilemmas. Journal of Experimental Social Psychology. 1989;25(4):287–313.

[8] Carpenter JP. Punishing free-riders: How group size affects mutual monitoring and the provision of public goods. Games and Economic Behavior. 2007;60(1):31–51.

[9] Agrawal A, Goyal S. Group size and collective action third-party monitoring in common-pool resources. Comparative Political Studies. 2001;34(1):63–93.

[10] Xu B, Cadsby CB, Fan L, Song F. Group size, coordination, and the effectiveness of punishment in the voluntary contributions mechanism: An experimental investigation. Games. 2013;4(1):89–105.

[11] Brewer MB, Kramer RM. Choice behavior in social dilemmas: Effects of social identity, group size, and decision framing. Journal of personality and social psychology. 1986;50(3):543.

[12] Homans GC. Social behavior: Its elementary forms New York: Harcourt Brace Jovanovich; [1961] 1974.

[13] Kelley HH, Thibaut JW. Interpersonal relations New York: Wiley; 1978.

[14] Van Lange PA. Collective rationality: The integrative model explains it (as) well. Acta psychologica. 2008;128(2):405–8. 10.1016/j.actpsy.2008.01.005 18316061

[15] Colman AM, Pulford BD, Rose J. Collective rationality in interactive decisions: Evidence for team reasoning. Acta psychologica. 2008;128(2):387–97. 17868630 10.1016/j.actpsy.2007.08.003

[16] Colman AM, Pulford BD, Rose J. Team reasoning and collective rationality: Piercing the veil of obviousness. Acta psychologica. 2008;128(2):409–12. 10.1016/j.actpsy.2008.04.001 18486930

[17] Bacharach M. Beyond individual choice: Teams and frames in game theory: Princeton University Press; 2006.

[18] Liebrand WB, McClintock CG. The ring measure of social values: A computerized procedure for assessing individual differences in information processing and social value orientation. European journal of personality. 1988;2(3):217–30.

[19] Grujić J, Eke B, Cabrales A, Cuesta JA, Sánchez A. Three is a crowd in iterated prisoner's dilemmas: Experimental evidence on reciprocal behavior. Scientific reports. 2012;2. 10.1038/srep00638 PMC3435562 22962633

[20] Yamagishi T. Factors mediating residual effects of group size in social dilemmas Japanese Journal of Psychology. 1990;61(3):162–9.

[21] Abele S, Stasser G, Chartier C. Conflict and coordination in the provision of public goods: A conceptual analysis of continuous and step-level games. Personality and Social Psychology Review. 2010;14(4):385–401. 10.1177/1088868310368535 20519698

[22] Van Lange PA. The pursuit of joint outcomes and equality in outcomes: An integrative model of social value orientation. Journal of personality and social psychology. 1999;77(2):337.

[23] Fehr E, Schmidt KM. A theory of fairness, competition, and cooperation. Quarterly journal of Economics. 1999:817–68.

[24] Gaube T. Group size and free riding when private and public goods are gross substitutes. Economics Letters. 2001;70(1):127–32.

[25] Marwell G, Ames RE. Experiments on the provision of public goods. I. Resources, interest, group size, and the free-rider problem. American Journal of Sociology. 1979;84(6):1335–60. 10.2307/2777895

[26] Nosenzo D, Quercia S, Sefton M. Cooperation in small groups: The effect of group size. Experimental Economics. 2013:1–11.

[27] Zhang X, Zhu F. Group size and incentives to contribute: A natural experiment at chinese wikipedia. The American economic review. 2011;101(4):1601–15.

[28] Haan M, Kooreman P. Free riding and the provision of candy bars. Journal of Public Economics. 2002;83(2):277–91.

[29] Lipford JW. Group size and the free-rider hypothesis: An examination of new evidence from churches. Public choice. 1995;83(3–4):291–303.

[30] Zelmer J. Linear public goods experiments: A meta-analysis. Experimental Economics. 2003;6(3):299–310.

[31] Isaac RM, Walker JM, Williams AW. Group size and the voluntary provision of public goods: Experimental evidence utilizing large groups. Journal of Public Economics. 1994;54(1):1–36.

[32] Fischbacher U, Gächter S, Fehr E. Are people conditionally cooperative? Evidence from a public goods experiment. Economics Letters. 2001;71(3):397–404.

[33] Kuhlman DM, Marshello AF. Individual differences in game motivation as moderators of preprogrammed strategy effects in prisoner's dilemma. Journal of Personality and Social Psychology. 1975;32(5):922 1185519 10.1037//0022-3514.32.5.922

[34] Smith ER, Conrey FR. Agent-based modeling: A new approach for theory building in social psychology. Personality and Social Psychology Review. 2007;11(1):87–104. 10.1177/1088868306294789 18453457

[35] Kerr NL, Kaufman-Gilliland CM. “. And besides, i probably couldn't have made a difference anyway”: Justification of social dilemma defection via perceived self-inefficacy. Journal of Experimental Social Psychology. 1997;33(3):211–30.

[36] Ostrom E. Collective action and the evolution of social norms. The Journal of Economic Perspectives. 2000:137–58.

[37] Lawler EJ, Yoon J. Commitment in exchange relations. American Sociological Review. 1996;61(1):89–108.

[38] Horne C. The enforcement of norms: Group cohesion and meta-norms. Social Psychology Quarterly. 2001:253–66.

[39] Lawler EJ. An affect theory of social exchange. American Journal of Sociology. 2001;107(2):321–52.

