# Supplementary material for: Dilemma of Dilemmas: How Collective and Individual Perspectives Can Clarify the Size Dilemma in Voluntary Linear Public Goods Dilemmas
Source: PLoS One. 2015 Mar 23;10(3):e0120379. doi: 10.1371/journal.pone.0120379 (PMC4370737; doi:10.1371/journal.pone.0120379)
Supplement: S1 Fig — Because multipliers are constrained from 1 < α < n, we have shown four values to cover the range of α when n = 2. (PDF) [file pone.0120379.s002.pdf]

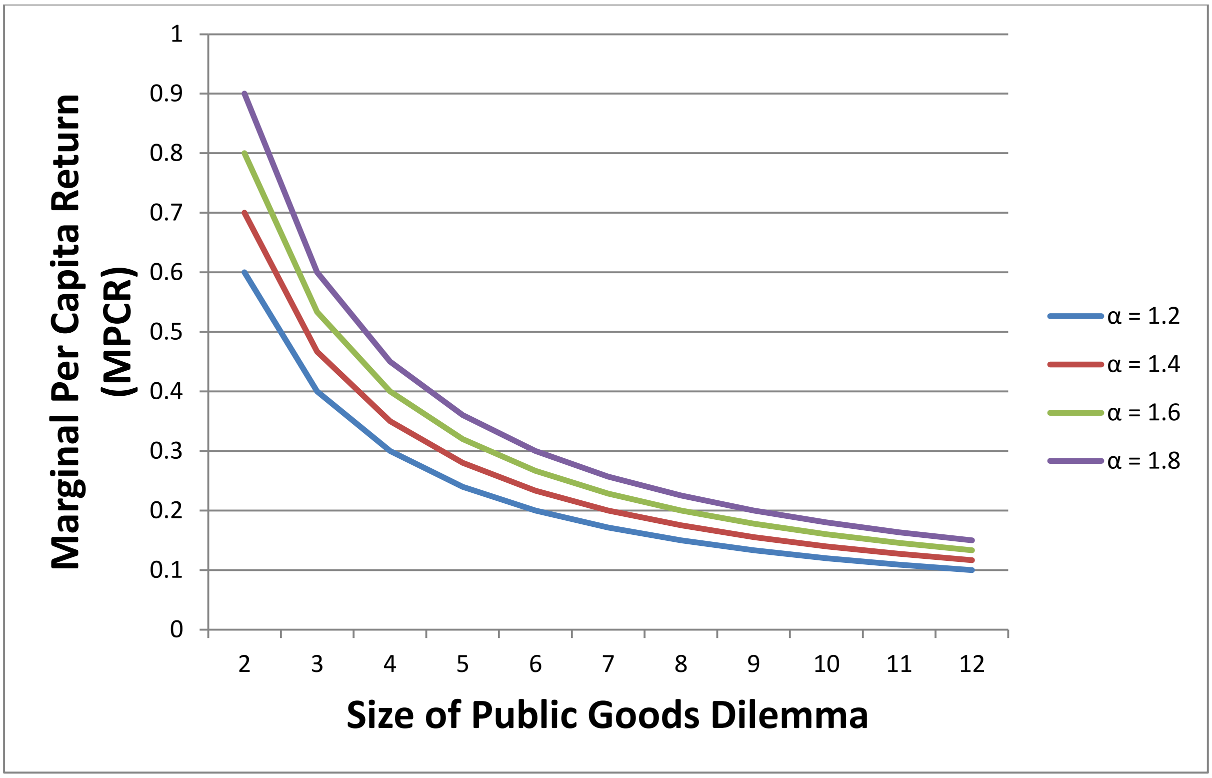

S1 Figure. Marginal Per Capita Return by different sizes of public goods dilemma and different multipliers (α). Because multipliers are constrained from $1 < \alpha < n$, we have shown four values to cover the range of α when n = 2.
