# Supplementary material for: Dilemma of Dilemmas: How Collective and Individual Perspectives Can Clarify the Size Dilemma in Voluntary Linear Public Goods Dilemmas
Source: PLoS One. 2015 Mar 23;10(3):e0120379. doi: 10.1371/journal.pone.0120379 (PMC4370737; doi:10.1371/journal.pone.0120379)
Supplement: S2 Fig — MPCR values are always between 0 and 1, but for a 2-person public goods dilemma, the MPCR must remain about .5, so we have shown four values to cover the range of .5 to 1. (PDF) [file pone.0120379.s003.pdf]

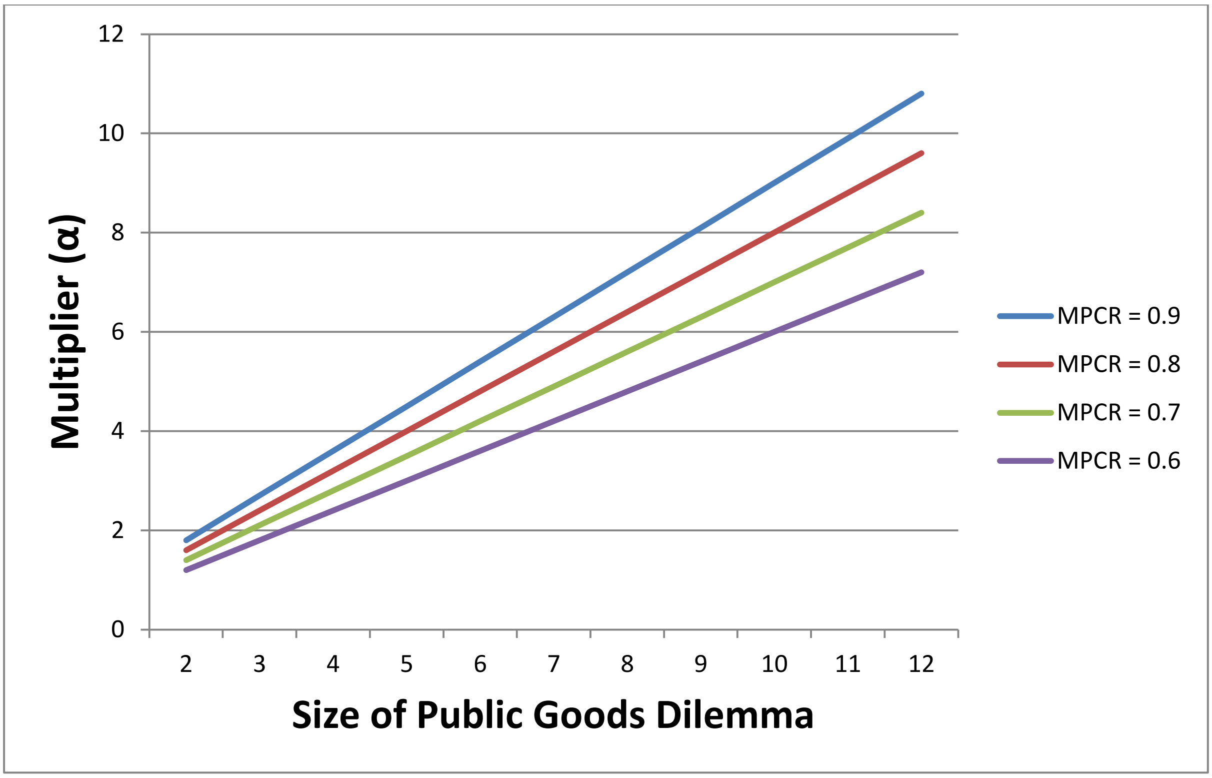

S2 Figure. Multiplier values by different sizes of public goods dilemma and different Marginal Per Capita Return values. MPCR values are always between 0 and 1, but for a 2-person public goods dilemma, the MPCR must remain about .5, so we have shown four values to cover the range of .5 to 1.
