# Supplementary material for: Dilemma of Dilemmas: How Collective and Individual Perspectives Can Clarify the Size Dilemma in Voluntary Linear Public Goods Dilemmas
Source: PLoS One. 2015 Mar 23;10(3):e0120379. doi: 10.1371/journal.pone.0120379 (PMC4370737; doi:10.1371/journal.pone.0120379)
Supplement: S3 Fig — Note that complete equality is 0. (PDF) [file pone.0120379.s004.pdf]

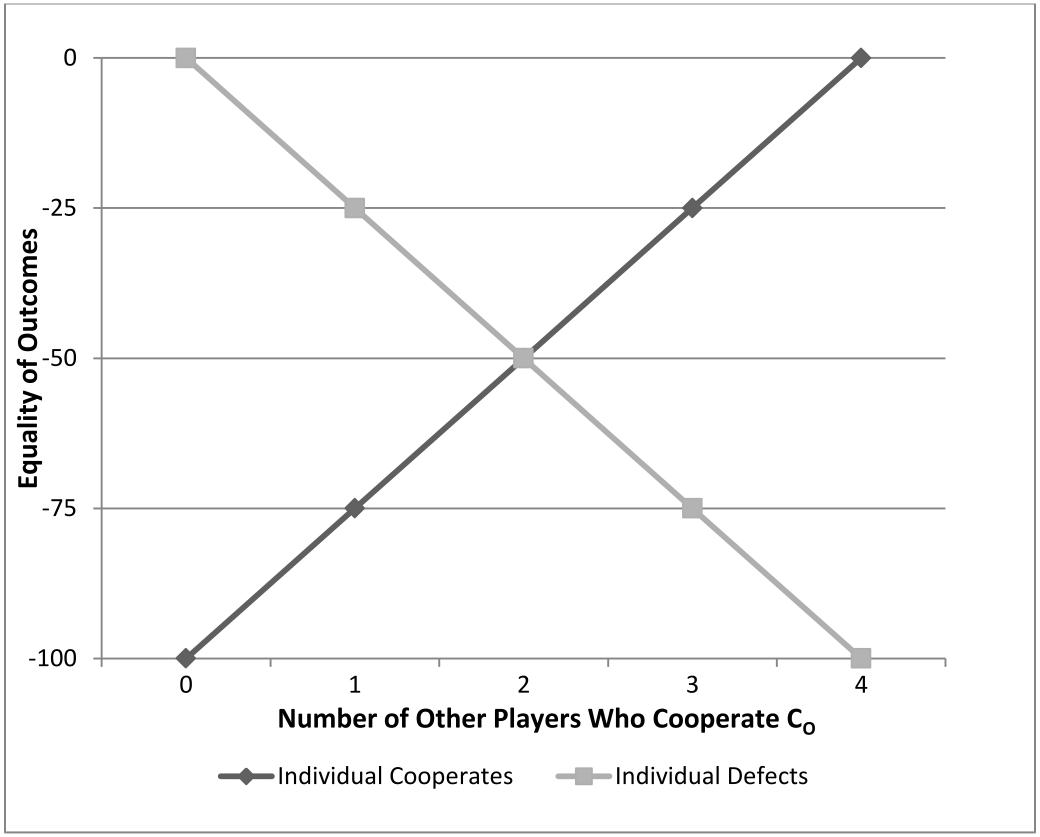

S3 Figure. *Equality of Outcomes* equation, $-\left|z(1 - c_S - \frac{n-1-c_O}{n-1})\right|$, for *n = 5, z = 100*, for varying if the individual cooperates, c_S, and how many other players cooperate, c_O. Note that complete equality is 0.
