# Supplementary material for: Dilemma of Dilemmas: How Collective and Individual Perspectives Can Clarify the Size Dilemma in Voluntary Linear Public Goods Dilemmas
Source: PLoS One. 2015 Mar 23;10(3):e0120379. doi: 10.1371/journal.pone.0120379 (PMC4370737; doi:10.1371/journal.pone.0120379)
Supplement: S1 File — (PDF) [file pone.0120379.s005.pdf]

S1 File: Missing Responses

Participants that missed 3 or more selections were timed out of the program and their entire group was excluded from our dataset. Of those included, 15 of the 390 participants (3.8%) missed a total of 21 rounds (0.2% of total rounds). By condition, they are listed as the first values in Table A. Additionally 9 participants missed the first round and 3 of those same participants also missed the second round, suggesting that they were not paying attention when the rounds began. The waiting room gave a countdown timer until they would definitely be assigned to a group, yet they could easily be assigned to a group prior to the timer expiring. Also participants were given twice as much time in the first and second round compared to other rounds. The numbers in brackets in Table A are the missed rounds 3-31 by condition. There was only one case in which multiple participants in the same group missed rounds, but it was both participants missing the first round.

Table A. Computer responses within the experiment.

	2-Person	5-Person	
		Multiplier Constant	MPCR Constant
Lower Values	2 [0]	2 [1]	5 [2]
Higher Values	8 [2]	4 [4]	0 [0]

Note: Computer responses in rounds 3-31 only in brackets
