# Supplementary material for: Dilemma of Dilemmas: How Collective and Individual Perspectives Can Clarify the Size Dilemma in Voluntary Linear Public Goods Dilemmas
Source: PLoS One. 2015 Mar 23;10(3):e0120379. doi: 10.1371/journal.pone.0120379 (PMC4370737; doi:10.1371/journal.pone.0120379)
Supplement: S2 File — (PDF) [file pone.0120379.s006.pdf]

S2 File: Genetic Algorithm Optimization

To determine optimal parameters for our agent-based simulation we created a genetic algorithm. The chromosomes included the following variables from versions of our agent-based simulation: W_1, W_2, W_3, population probability of a *Random Behavior* for each agent per round, *Adjusted Weight Probability* for each individual (the SD of a random normally distributed term, M=0, added to each weight for each individual agent), population *Utility Function Probability* (SD of a random normally distributed term, M=0, added to the utility function every time it is used), and *Memory*, W_M, according to the temporal utility function $U_{M,t} = (W_M)(U_t) + (1\text{-}W_M)(U_{M,t\text{-}1})$. The weights ($W_1$, W_2, W_3) were allowed to vary independently for purposes of the genetic algorithm but are scaled to $W_1 + W_2 + W_3 = 1$ for reporting. The chromosomes were all initialized randomly from 0 to 1, with the *Utility Function Probability* multiplied by 400 for the public goods dilemmas to obtain the range of 0 to 400 (the value of full cooperation for the most beneficial dilemma). While the chromosomes were initialized from 0 to 1, there values were not constrained, except when negative numbers could not be used (e.g., standard deviations) the chromosome values were constrained to positive numbers.

On each generation members of the population of potential solutions were each used as parameters for 100 public goods dilemma simulations for each of the six experimental conditions. To evaluate the quality of solution, the fitness function compared the average cooperation on those 100 runs with the empirical level of cooperation in the same experimental condition. The fitness of a possible solution was the Euclidian distance from the simulated cooperation level to the empirical cooperation level across all six experimental conditions. For each generation, fitness was computed for all members, the half with the best (lowest) fitness survived into the next generation. The other half was replaced with new members. A new member was formed by randomly selecting two parents from the survivors, and then randomly selecting the chromosome of one or the other. For each new member, one randomly selected chromosome was mutated by adding a random value to it (M=0; SD = .1). This meant that the mutated values were not constrained to existing values, but they were more likely to be values near the parents' value. Note that the method of leaving a member in as long as it is in the top half of the population and reevaluating its fitness each generation means that if a member received by chance a more favorable fitness score on one generation, they would be unlikely to receive it again on subsequent generations. Leaving parents in the population and reevaluating their fitness are both techniques to ensure adequate searching of desirable solution spaces without converging to fast to local optima.

We conducted 10 genetic algorithm trials. Each had a population of 50 members and stopped after 50 generations, according to preliminary tests. The results indicate that in all trials, the population of solutions converged to 0 or a negligible amount above 0 for the *Adjusted Weight Probability* and *Utility Function Probability* terms, so these are not discussed further. Two of the 10 trials produced sub-optimal solutions indicated by no potential solutions with fitnesses < .10. Another trial had only one solution with a fitness < .10 at .090 suggesting that the genetic algorithm had failed to converge on a solution by the 50th generation.

The remaining seven trials converged (meaning multiple members reach the same solution which produced good fitness scores (< .10)), so we examine the possible solutions with the best (lowest) fitness in each genetic algorithm trial (Table B). One solution, produced on three trials, had low random behavior (.121) and weights (.213, .479, .309, respectively) similar to our previously derived optimal solution (random behavior of .10 and weights of .2, .5, .3, respectively). Interestingly, the memory term was extremely low indicating the mixed motive agents considered the more distal past with almost as much weight as the immediate past (the previous terms would be weighted at .084, the one before it at .075 (.084 *(1 -.084)) and so forth), or a 10.4% decrease in weight per round. The other solution has much higher random behavior (.362) and weights that reflected more self-interest and less equality than the first solution (.365, .442, .193, respectively).

This solution was near unity for Memory indicating a negligible influence for any round except the immediate predecessor.

Our assessment is that with the high levels of random behavior the more distal past provides less information on how to respond, thus reducing the necessity of memory. Additionally, both random behavior and equality of outcomes push agents toward behaving more moderately (e.g., more equal levels of cooperation and defection), so it is not surprising that lower levels of one are associated with higher levels of the other. Both solutions obtain integrative model weights fairly near our previously found solution, with the low random solution obtaining extremely similar weights. We feel confident that the pattern of the importance of equality in the integrative model equation is again confirmed by this genetic algorithm exercise. The importance of memory, however, poses new and interesting questions for these models and should be explored in future research.

Table B. Mean of best fitness solutions from the seven genetic algorithm trials that converged to near optimal solutions.

	Trials	Fitness	Random Behavior	Memory	W_1	W_2	W_3
Low Random Behavior Solution	3	.064	.121	.084	.213	.479	.309
		[.048-.073]	[.117-.127]	[.027 -.114]	[.203 - .218]	[.475 - .484]	[.305 - .312]
High Random Behavior Solution	4	.052	.362	1.021	.365	.442	.193
		[.041 - .059]	[.329 - .369]	[.907 – 1.088]	[.328 - .404]	[.431 - .463]	[.165 - .231]

Note: Range in brackets.
