# Supplementary material for: Dilemma of Dilemmas: How Collective and Individual Perspectives Can Clarify the Size Dilemma in Voluntary Linear Public Goods Dilemmas
Source: PLoS One. 2015 Mar 23;10(3):e0120379. doi: 10.1371/journal.pone.0120379 (PMC4370737; doi:10.1371/journal.pone.0120379)
Supplement: S1 Table — (PDF) [file pone.0120379.s007.pdf]

S1 Table

Robustness checks for the simulated results: the models that best match the empirical result pattern

	2-Person		5-Person				Weights		
			Multiplier Constant		MPCR Constant				
	Low Benefit	High Benefit	Low Benefit	High Benefit	Low Benefit	High Benefit	W_1	W_2	W_3
Empirical Results	.354	.629	.162	.237	.602	.611			
Simulated Results									
With initial parameters	.248	.616	.089	.308	.618	.644	.2	.5	.3
With no defectors	.497	.936	.176	.801	.932	.936	.2	.5	.3
With random utility term per round[a]	.349	.613	.090	.176	.596	.635	.2	.5	.3
With random utility term per round[a]	.255	.466	.217	.290	.592	.645	.1	.5	.4
With random utility term per round[a]	.459	.657	.390	.502	.628	.631	0	.6	.4
With random utility term per round[a]	.561	.627	.288	.456	.632	.632	.1	.6	.3
With random weights term per person	.259	.634	.079	.295	.616	.634	.2	.5	.3
With temporal memory (W_M=.8)	.415	.640	.088	.220	.621	.644	.2	.5	.3

[a] There were four weights that matched the empirical pattern, however W_1=.2, W_2=.5, W_3=.3 was the best match.
